# Prostate Diffusion-weighted Imaging: Selecting the Optimal Repetition Time Using Synthetic Imaging Techniques

**DOI:** 10.2463/mrms.tn.2025-0101

**Published:** 2026-01-15

**Authors:** Atsushi Higaki, Tsutomu Tamada, Mitsuru Takeuchi, Yu Ueda, Yuichi Kojima, Takuma Maruhisa, Hiroyuki Watanabe, Kazunori Moriya, Yoshihiko Fukukura, Akira Yamamoto

**Affiliations:** 1 Department of Radiology, Kawasaki Medical School, Kurashiki, Okayama, Japan; 2 Department of Radiology, Radiolonet Tokai, Nagoya, Aichi, Japan; 3 Philips Japan, Tokyo, Japan

**Keywords:** diffusion-weighted imaging, prostate cancer, repetition time

## Abstract

The optimal TR for prostate diffusion-weighted imaging (DWI) remains unclear. Given prostate cancers’ shorter T1/T2 relaxation times versus benign tissues, TR adjustment may improve contrast. We evaluated 56 clinically significant cancers in 33 patients, comparing synthetic DWI (b-value = 2000 s/mm^2^) at TR500/1000/1500/2000 ms against conventional TR6000 ms. Assessments included contrast ratio, apparent SNR, lesion conspicuity score, DWI scores based on Prostate Imaging Reporting and Data System (PI-RADS) v2.1, and background suppression. TR6000 showed significantly lower contrast ratio but higher apparent SNR compared with shorter TRs. TR1000–2000 showed higher lesion conspicuity score than TR500, while TR1500–6000 had higher DWI scores than TR500. TR500–1500 provided better background suppression than TR2000/6000. For optimal balance of contrast and noise, TR1000 or TR1500 is recommended for prostate DWI, potentially enhancing prostate cancer detection in clinical practice.

## Introduction

The European Society of Urogenital Radiology guidelines recommend including diffusion-weighted imaging (DWI) as an essential component of multiparametric MRI (mp-MRI) to detect clinically significant prostate cancer (csPCa).^1^^,^^2^ DWI plays a critical role in diagnosing PCa.^2^^,^^3^ The primary role of mp-MRI is to identify csPCa, thereby reducing unnecessary biopsies, avoiding overdiagnosis of low-risk lesions, and improving risk stratification.^4^^–^^8^ High b-value DWI (b ≥ 1400 s/mm^2^) and apparent diffusion coefficient (ADC) maps are particularly valuable for differentiating malignant from benign prostate tissues.

Prostate cancer is characterized by shorter T1 and T2 relaxation times than those of benign prostate tissue.^9^^–^^14^ Based on this property, using a shorter TR may enhance the T1 shine-through effect and improve the contrast between tumor and background tissue on DWI.^15^^–^^17^ However, conventional DWI—typically acquired with a long TR—suppresses T1 contrast and may not adequately distinguish PCa from benign tissue.^9^^,^^13^^,^^15^^–^^17^

While short-TR DWI could improve diffusion contrast using T1 shine-through effect, its diagnostic utility remains unclear, and the optimal TR has not been established. Furthermore, a shorter TR may reduce the apparent SNR (aSNR), requiring a careful assessment that balances contrast enhancement with image quality.^18^

In this study, we aimed to determine the optimal TR for prostate DWI using synthetic DWI (SyDWI), a technique we developed.^16^ SyDWI enables pure comparison of different TR values from a single scan, without confounding effects from changes in positioning, motion, acquisition parameters, or imaging artifacts. We evaluated tumor contrast ratio (CR), aSNR, lesion conspicuity score (LCS), the DWI score used in Prostate Imaging Reporting and Data System (PI-RADS) assessment, and background signal suppression. Ultimately, we aimed to identify the most suitable TR for prostate DWI, thereby enhancing diagnostic accuracy in detecting prostate cancer.

## Materials and Methods

### Study population

This study was approved by the Institutional Review Board of Kawasaki Medical School Hospital (Approval number: 5946). Informed consent was waived through an opt-out form posted on the institution’s website. The study included patients who underwent 3.0T MRI between November 2021 and August 2022 for suspected prostate cancer and were subsequently diagnosed with csPCa following radical prostatectomy. csPCa was defined as a Gleason score ≥ 7 with a tumor diameter ≥ 5 mm, or a Gleason score of 3 + 3 with a tumor volume ≥ 0.5 mL (corresponding to a tumor diameter ≥ 8 mm). Tumor size was measured using the mp-MRI sequence that best depicted the lesion.

### Imaging protocol

All MR examinations were performed using a 3.0T scanner (Ingenia Elition 3.0T; Philips Healthcare, Best, the Netherlands) with an anterior and built-in posterior coil. To suppress bowel peristalsis, either 20 mg of scopolamine butylbromide (Buscopan; Boehringer Ingelheim Pharmacy, Yamagata, Japan) or 1 mg of glucagon (Glucagon-G Novo; Eisai Pharmacy, Tokyo, Japan) was administered intramuscularly before imaging was conducted. The imaging sequences included DWI, T2-weighted imaging, T1-weighted imaging, 3D T2-weighted imaging, and dynamic contrast-enhanced MRI. In the present study, evaluation was primarily based on DWI.

### DWI acquisition

DWI was acquired using a spin echo–based single-shot echo-planar imaging sequence. In SyDWI, 2 different sets of TR and TE were used for b0 images, while b2000 images were acquired with a single TR and TE, mirroring conventional protocols. This approach is termed “Multiple Repetition Time Multiple Echo Time DWI (MRME DWI).” Short-TR and long-TR scans were performed separately. Because initial short-TR images can exhibit elevated signal intensities, a dummy scan was performed before data acquisition to minimize this effect.

To accommodate variable TE settings, we used a dual-echo acquisition method, capturing all data in a single scan. Identical bandwidths were maintained across TEs. The reference standard was our institutional clinical DWI protocol (“conventional DWI”).

Table 1 presents a list of the detailed imaging parameters for both SyDWI and conventional DWI. All scans were performed in a single imaging session.

### Construction of SyDWI using MRME DWI

SyDWI was calculated on a pixel-by-pixel basis from MRME DWI data, which includes b0 images acquired with 2 TR and TE combinations, and b2000 images acquired with a single TR and TE.

The signal intensity (SI) of spin-echo (SE) DWI is given by:

(1) $$SI=\alpha \cdot PD\cdot (1-e^{-TR/T1})\cdot e^{-TE/T2}\cdot e^{-b\cdot D}$$

where α is a proportionality constant and PD represents proton density.

T1 was estimated from b0 images acquired at different TRs with a fixed TE, while T2 was derived from b0 images acquired at different TEs with a long and fixed TR.

To calculate the term [α ∙ PD ∙ e^(‒b∙D)] for b = 2000 s/mm^2^, we used the signal intensity from DWI acquired at TR = 6000 ms and TE = 70 ms, and rewrote equation (1) as:

(2) $$\alpha \cdot PD\cdot e^{-b\cdot D}=SI\;\text{in}\;DWI\text{/}[(1-e^{-TR/T1})\cdot e^{-TE/T2}]$$

DWI at any desired TR and TE for each acquired b-value was then generated using the equation (Fig. 1).

All post-processing was performed on the MR console using the Philips Research Imaging Development Environment. In this study, SyDWI at b = 2000 s/mm^2^ was generated at 4 TR values (500, 1000, 1500, and 2000 ms) using Philips Research Imaging Development Environment. Since PCa exhibits a lower T2 than that of benign prostate tissues, the TE of SyDWI was set to 0 ms to fully eliminate the T2 shine-through effect.

### Image analysis

Image analysis was performed using SYNAPSE Enterprise-PACS (FUJIFILM Medical). The target lesion was defined as the tumor with the highest Gleason score confirmed histopathologically; when multiple lesions showed the same score, the largest lesion was selected. On the slice containing the maximum diameter of the selected lesion, a circular or elliptical ROI was placed. A matching ROI was placed on the contralateral, pathologically confirmed normal prostate tissue within the same slice, using the same size as the tumor ROI. ROI placement was conducted by 2 radiologists with 1 and 16 years of experience in prostate MRI interpretation, and the final decision was made through consensus evaluation.

### Quantitative and qualitative evaluation

#### Quantitative evaluation

The tumor CR was calculated by dividing the signal intensity of the tumor ROI by that of the benign peripheral zone tissue ROI. The aSNR was defined as the signal intensity of the benign peripheral zone divided by the standard deviation of the signal in the internal obturator muscle. Benign prostatic tissues were histologically confirmed following total prostatectomy.

#### Qualitative evaluation

Two radiologists independently performed the qualitative evaluation: Reader 1 with 25 years and Reader 2 with 2 years of experience in prostate MRI interpretation. LCS was rated on a 5-point scale: 0 (low), 1 (isointense), 2 (slightly high), 3 (moderately high), and 4 (very high). The DWI score was assigned using PI-RADS v2.1 on a 1–5 scale, with reference to the corresponding ADC map. This ADC map was generated from a separate clinical DWI acquisition obtained with TR/TE = 6000/70 ms and b-values of 0 and 1000 s/mm^2^, and the same map was used consistently for scoring all syDWI TR conditions as well as conventional DWI (b = 2000 s/mm^2^). Prostate background signal was graded as 0 (very high), 1 (moderately high), 2 (mildly high), 3 (slightly high), or 4 (low).

#### Statistical analysis

All statistical analyses were performed using SPSS version 24.0 (SPSS, Chicago, IL, USA) and JMP version 17.0.0 (SAS, Cary, NC, USA). The Kruskal–Wallis test was used to evaluate overall differences among TR conditions. If a significant difference was found, the Mann–Whitney U test with Bonferroni correction was applied for pairwise comparisons. A P-value < 0.005 was considered statistically significant. Spearman’s rank correlation coefficient (ρ) was employed to assess the monotonic relationships between TR and CR, and between TR and aSNR. A 2-sided P-value of less than 0.05 was considered statistically significant.

## Results

### Patient selection

In total, 35 patients suspected of having prostate cancer were enrolled, all of whom underwent MRI, including sequences required for SyDWI. One patient was excluded owing to incomplete image acquisition of the b0 sequences, and another was excluded because the lesion identified on pathology could not be visualized on MRI. Ultimately, 33 patients (56 lesions) were included in the analysis.

### Baseline characteristics of patients and lesions

Table 2 shows a summary of the baseline characteristics of the participants and their lesions. The median age was 69.5 years (interquartile range [IQR], 64.0–74.0). The median total prostate-specific antigen (T-PSA) was 7.86 ng/mL (IQR, 5.46–11.04), and the median PSA density was 0.30 ng/mL/cm^3^ (IQR, 0.24–0.48).

Regarding lesion distribution, 31 lesions (55.4%) were in the peripheral zone, 22 (39.3%) in the transition zone, and 3 (5.4%) involved both zones. The median tumor ADC value was 0.75 ×10^−3^ mm^2^/s (IQR, 0.68–0.84), and the median tumor size was 13.6 mm (IQR, 9.1–17.9).

By PI-RADS classification, 5 lesions (8.9%) were PI-RADS 3, 32 (57.1%) were PI-RADS 4, 18 (32.1%) were PI-RADS 5, and 1 (1.8%) was not assessed using PI-RADS because of atypical imaging features, including cystic and hemorrhagic components, in a pathologically confirmed ductal adenocarcinoma, for which PI-RADS is considered to have limited applicability. Pathological T stages were: T2a in 10 (17.9%), T2c in 20 (35.7%), T3a in 24 (42.9%), and T3b in 2 lesions (3.6%).

### Influence of different TRs on imaging evaluation

#### Quantitative evaluation

#### Tumor CR

CR at TR6000 (2.2 ± 0.7) was significantly lower than at TR500 (8.0 ± 7.2), TR1000 (6.8 ± 4.7), TR1500 (6.2 ± 3.8), and TR2000 (5.9 ± 3.4) (*P* < 0.005). No statistically significant differences were observed among the TR500–TR2000 conditions, which exhibited high and comparable contrast.

Figure 2 presents an illustration of these results, clearly showing the substantial reduction in CR at TR6000 compared with that at other TR values. Furthermore, Spearman’s rank correlation analysis revealed a moderate negative correlation between CR and TR, with a Spearman’s ρ of –0.479 (*P* < 0.001), indicating that CR decreases as TR increases.

#### aSNR

The aSNR at TR6000 (19.4 ± 5.6) was significantly higher than that at TR500 (2.8 ± 1.1), TR1000 (2.9 ± 1.5), TR1500 (3.1 ± 1.6), and TR2000 (3.4 ± 1.6) (*P* < 0.001). Additionally, the aSNR at TR2000 (3.4 ± 1.6) was significantly higher than that at TR500 (2.8 ± 1.1) (*P* = 0.034). No other statistically significant differences in aSNR were observed among the remaining conditions.

Figure 3 illustrates these results, clearly showing a substantial increase in aSNR at TR6000 compared with that at the other TR values. Furthermore, Spearman’s rank correlation analysis revealed a moderate positive correlation between aSNR and TR, with a Spearman’s ρ of 0.563 (*P* < 0.001), indicating that aSNR increases as TR increases.

#### Qualitative evaluation

Table 3 shows a summary of the detailed qualitative results for LCS, DWI score, and background signal suppression across the different TR conditions.

#### Lesion conspicuity score

LCS values for TR1000 (Reader 1, 3.8 ± 0.5; Reader 2, 3.8 ± 0.4), TR1500 (Reader 1, 3.9 ± 0.4; Reader 2, 3.8 ± 0.4), TR2000 (Reader 1, 3.8 ± 0.4; Reader 2, 3.8 ± 0.4), and TR6000 (Reader 1, 3.8 ± 0.4; Reader 2, 3.8 ± 0.5) were significantly higher than those for TR500 (Reader 1, 3.3 ± 0.6; Reader 2, 3.4 ± 0.7) (*P* < 0.005), except for the comparison between TR500 and TR1000 in Reader 2, which was not significant. However, no significant differences were observed among TR1000, TR1500, TR2000, and TR6000 (Reader 1, 3.8 ± 0.4; Reader 2, 3.8 ± 0.5) for either reader (Table 3).

#### DWI score

The DWI score at TR2000 (Reader 1, 3.9 ± 0.5; Reader 2, 4.0 ± 0.4) and TR6000 (Reader 1, 3.9 ± 0.3; Reader 2, 3.9 ± 0.5) was significantly higher than that at TR500 (Reader 1, 3.6 ± 0.6; Reader 2, 3.7 ± 0.7) for both readers (*P* < 0.005). TR1500 also yielded a significantly higher DWI score (Reader 1, 3.9 ± 0.4) than TR500 in Reader 1 (*P* < 0.005). However, no significant differences were observed among TR1000 (Reader 1, 3.9 ± 0.5; Reader 2, 3.9 ± 0.4), TR1500, TR2000, and TR6000 (Reader 1, 3.9 ± 0.3; Reader 2, 3.9 ± 0.5) for either reader (Table 3).

#### Background signal suppression

For both readers, background signal suppression scores at TR500 (Reader 1, 3.7 ± 0.6; Reader 2, 3.7 ± 0.6), TR1000 (Reader 1, 3.6 ± 0.6; Reader 2, 3.8 ± 0.6), TR1500 (Reader 1, 3.6 ± 0.6; Reader 2, 3.8 ± 0.4), and TR2000 (Reader 1, 3.4 ± 0.6; Reader 2, 3.8 ± 0.4) were significantly higher than those at TR6000 (Reader 1, 1.1 ± 0.8; Reader 2, 1.4 ± 0.5) (*P* < 0.005). Furthermore, Reader 1 found that the suppression score at TR500 was significantly higher than that at TR2000 (*P* < 0.005).

Figure 4 presents a representative case comparing conventional DWI with synthetic DWI at various TRs, clearly illustrating the effect of TR settings on lesion visualization.

## Discussion

In this study, we examined the optimal TR for prostate DWI using a novel SyDWI approach. This method allows simultaneous generation of conventional and synthetic images within a single acquisition session. Our findings revealed 3 key observations: first, CR values were significantly lower at TR6000 than at shorter TRs, and a statistically significant negative correlation was observed between TR and CR (Spearman’s ρ = –0.479, *P* < 0.0001), indicating that shorter TRs enhance tumor-to-background contrast. Second, aSNR showed a statistically significant positive correlation with TR (Spearman’s ρ = 0.563, *P* < 0.001), indicating that aSNR increases as TR becomes longer. TR6000 showed the highest aSNR, and TR2000 had significantly higher aSNR than TR500. However, the aSNR did not significantly differ among TR1000, TR1500, and TR2000. Third, TR1000 and TR1500 yielded superior results based on the comprehensive assessment of lesion conspicuity, PI-RADS scores, and background suppression. These findings directly address the study’s objective of identifying a TR setting that optimally balances contrast enhancement and noise suppression for effective prostate cancer visualization.

The inverse correlation between TR and CR underscores the importance of T1 contrast in enhancing lesion visibility on prostate DWI. Prostate cancers typically exhibit shorter T1 relaxation times than those of benign tissues, resulting in greater T1-weighted signal differences at shorter TRs.^9^^,^^15^

Conventional DWI protocols often use long TRs (e.g., TR6000), which suppress T1 effects and reduce tumor-to-background contrast. In contrast, SyDWI enables flexible TR modulation without extending scan time, allowing more prominent T1 shine-through effects.^9^^,^^10^ In this study, although CR differences among TR conditions ranging from 500 to 2000 ms were not statistically significant, the values showed a trend toward higher CR at shorter TRs. This supports the theoretical expectation that shorter TRs enhance T1 contrast and highlights SyDWI advantage in capturing this relationship. However, this contrast enhancement must be interpreted in the context of aSNR. In this study, excessively short TRs (e.g., TR500) resulted in significantly lower aSNR, potentially compromising diagnostic utility despite improved contrast.

In addition to CR findings, TR1000 and TR1500 showed the best results for LCS, DWI scores, and background signal suppression. TR1000, TR1500, and TR2000 had significantly higher LCS values than those of TR500, with no significant differences among TR1000–6000. Similarly, DWI scores were significantly higher for TR1500, TR2000, and TR6000 than for TR500, with no significant differences among TR1000–6000. Background signal suppression was more effective at TR500–1500, while TR2000 and TR6000 showed weaker suppression. Regarding aSNR, the TR6000 yielded the highest values, and the TR2000 was significantly higher than those of the TR500. No significant differences in aSNR were observed among TR1000, TR1500, and TR2000. These findings suggest that while TR500 provides excellent suppression and the highest CR, its overall image quality was perceived as inferior. This perceived degradation, likely due to a coarser noise texture not fully captured by ROI-averaged aSNR values, outweighed the benefit of high contrast and resulted in lower LCS and DWI score.^18^ In contrast, TR1000 and TR1500 balanced enhanced T1 contrast and acceptable noise levels, maintaining high diagnostic quality. Although TR2000 showed comparable LCS, DWI score, and aSNR to TR1000 and TR1500, background signal suppression tended to be weaker than that observed at shorter TRs, with a significant difference between TR500 and TR2000 for Reader 1. Considering the overall balance among lesion contrast, background suppression, and aSNR, TR1000 and TR1500 provided the most stable image quality for diagnostic use. However, since the difference from TR2000 was small, depending on scanner performance and noise characteristics, TR2000 may also be considered as a reasonable alternative. This trend aligns with study of Higaki et al., who investigated conventional (non-synthetic) short-TR DWI and reported improved prostate cancer visualization at TR1850 without compromising SNR.^15^ Thus, depending on scanner capabilities and imaging parameters, the optimal TR settings identified in this study (TR1000 and TR1500) may also be applicable to conventional (non-synthetic) DWI protocols, provided that image quality is sufficient to preserve aSNR and ensure that ADC values remain unaffected.

Prostate cancer has a distinctly lower T1 relaxation time than that of normal prostate tissue, a feature not typically seen in other malignancies.^9^^,^^13^^,^^15^^,^^17^ This makes optimizing TR in prostate DWI particularly important. However, such optimization has been challenging, as conventional DWI requires separate scans for each TR, leading to extended imaging times and logistical burdens. Consequently, comparative TR studies have been limited. The SyDWI technique used in this study addresses these limitations by generating multiple TR-specific images from a single acquisition, enabling direct comparison under consistent imaging conditions. This advancement facilitates efficient TR optimization and may improve standardization and diagnostic quality in prostate MRI. Furthermore, SyDWI has broader potential, including evaluations of other imaging parameters (e.g., b-value, TE) and extension to other organs, offering greater flexibility in DWI protocol design.^16^ In this study, TR1000 and TR1500 were identified as optimal parameters for prostate DWI, providing balanced performance across all evaluated image quality metrics.

However, some limitations should be noted. First, this was a single-institution study limited to cases of csPCa, and the applicability to lower-risk cancers or benign lesions remains unclear. Second, SyDWI relies on synthetic signal reconstruction, which may not fully replicate the noise characteristics of actual acquired DWI images. Interobserver agreement for the qualitative scores was not evaluated. Furthermore, we evaluated only a single b-value (b = 2000 s/mm^2^), leaving the interaction with other b-values unexplored. Finally, this study did not examine the diagnostic performance of SyDWI using the identified optimal TR settings. Specifically, because our cohort was limited to patients with confirmed csPCa, we could not directly evaluate the impact of the proposed TR settings on the risk of false negatives. However, our finding that shorter TRs improved tumor contrast suggests that this approach could potentially enhance lesion detectability, thereby reducing this risk. To validate and generalize these findings, future multicenter studies are needed to evaluate the diagnostic performance of this technique through direct comparison with conventional DWI in large cohorts of patients with suspected csPCa.

## Conclusion

Our findings reveal that TR1000–1500 ms provides optimal performance for prostate DWI. These findings may contribute to the optimization of prostate MRI protocols and improve diagnostic accuracy in prostate cancer detection.

## Figures and Tables

**Fig. 1:**
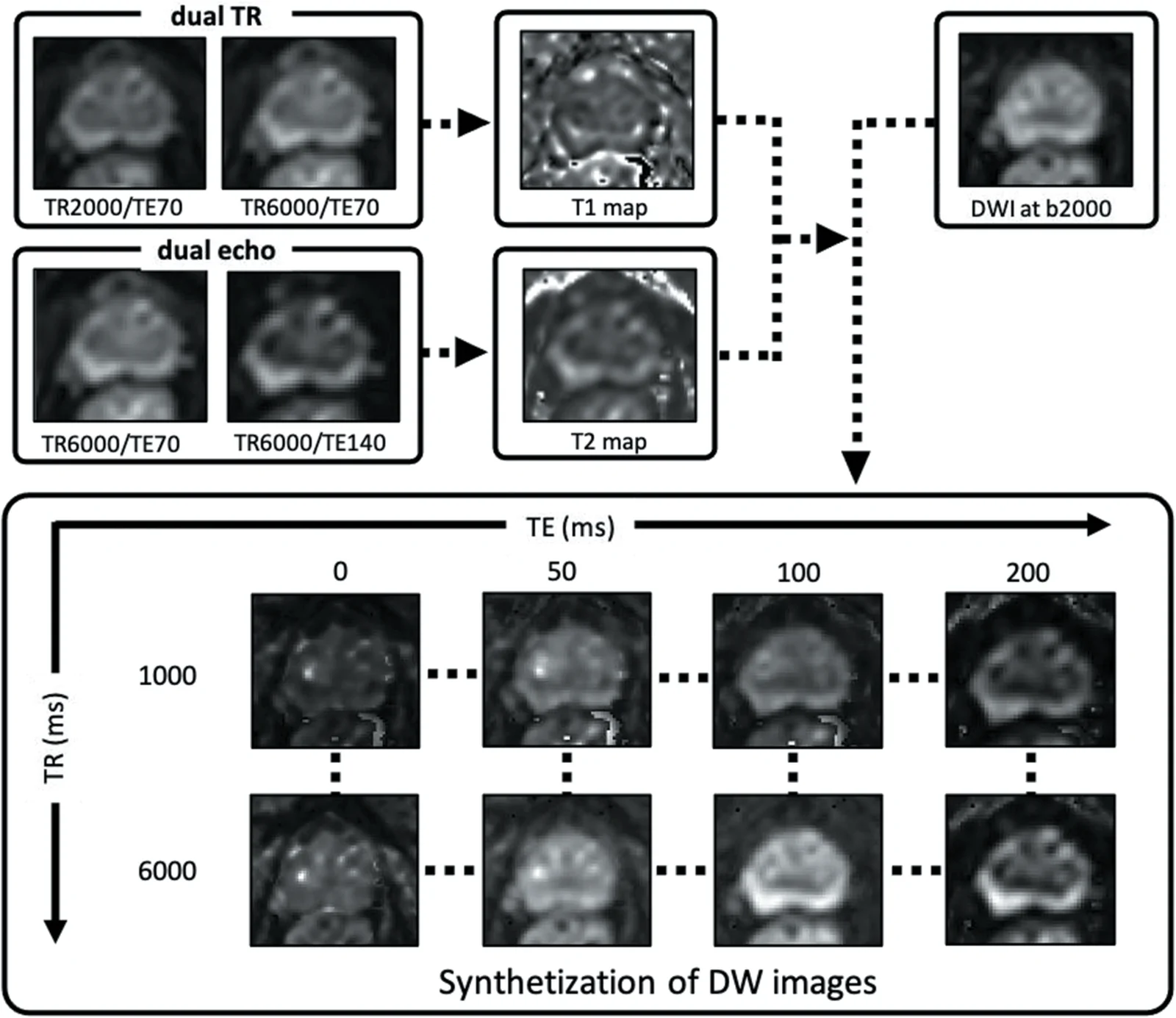
Flowchart of synthetic DWI computation based on MRME DWI data. This figure presents a schematic representation of the process used to generate SyDWI from MRME DWI data. The diagram outlines the following steps: first, b0 images are acquired at 2 different TRs (short and long) with a fixed TE and at 2 different TEs with a fixed long TR to estimate T1 and T2; next, DWI at b = 2000 s/mm^2^ is acquired with a single TR and TE to derive the term [α∙PD∙e^(‒b∙D)]; finally, SyDWI is computed at any desired TR and TE based on these parameters. MRME, multiple repetition time multiple echo time; SyDWI, synthetic diffusion-weighted imaging.

**Fig. 2:**
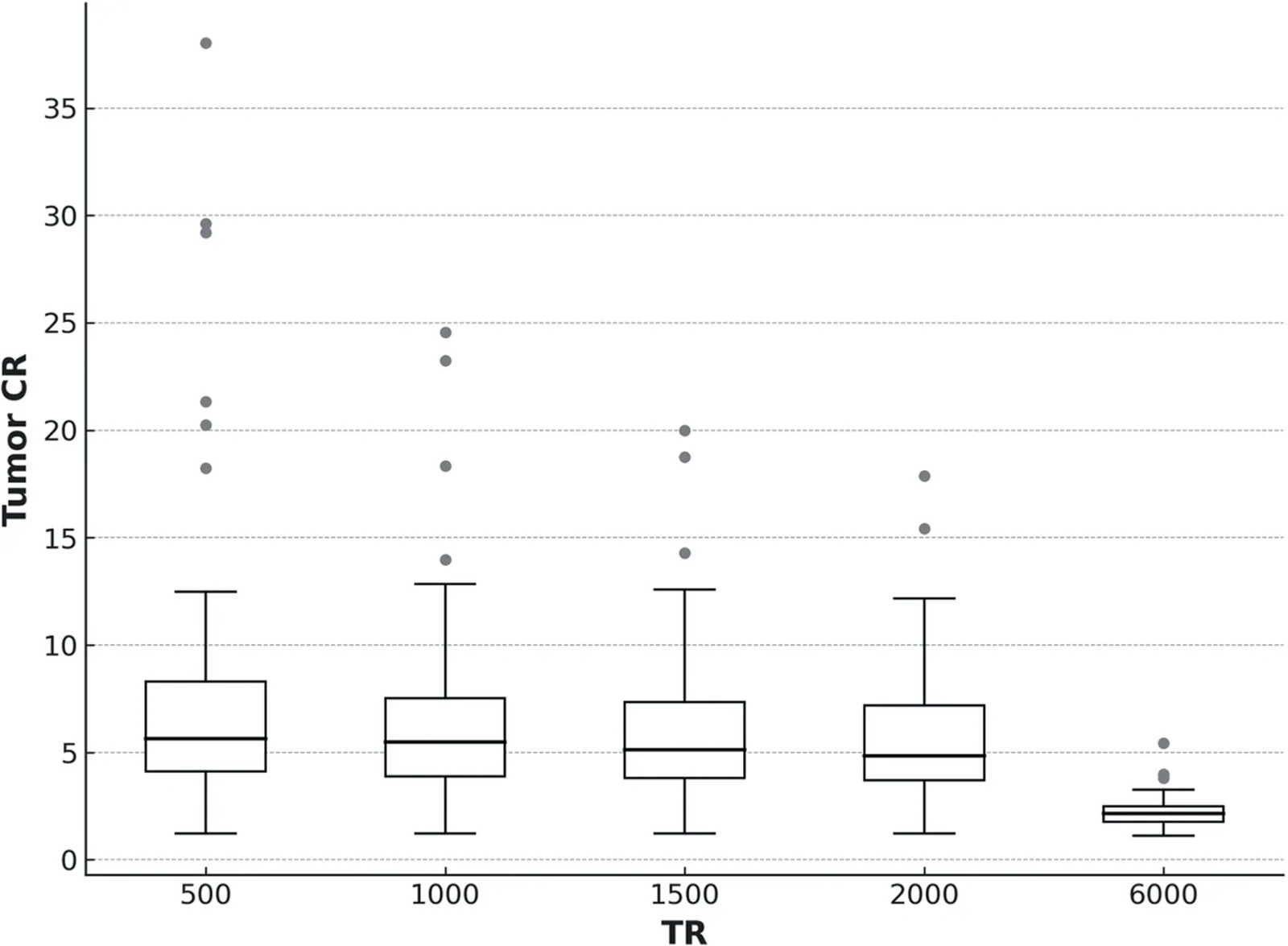
Box plot of tumor CR by TR condition. Box plots illustrate the distribution of the tumor CR obtained from DWI with b = 2000 s/mm^2^ at various repetition times (TR = 500, 1000, 1500, 2000, and 6000 ms). Tumor CR was calculated as the ratio of lesion SI to benign tissue SI. The CR at TR6000 was significantly lower than that at TR500, TR1000, TR1500, and TR2000 (*P* < 0.005). No statistically significant differences were observed among the CR values for TR500, TR1000, TR1500, and TR2000, which consistently exhibited relatively high contrast values. CR, contrast ratio, DWI, diffusion-weighted imaging; SI, signal intensity.

**Fig. 3:**
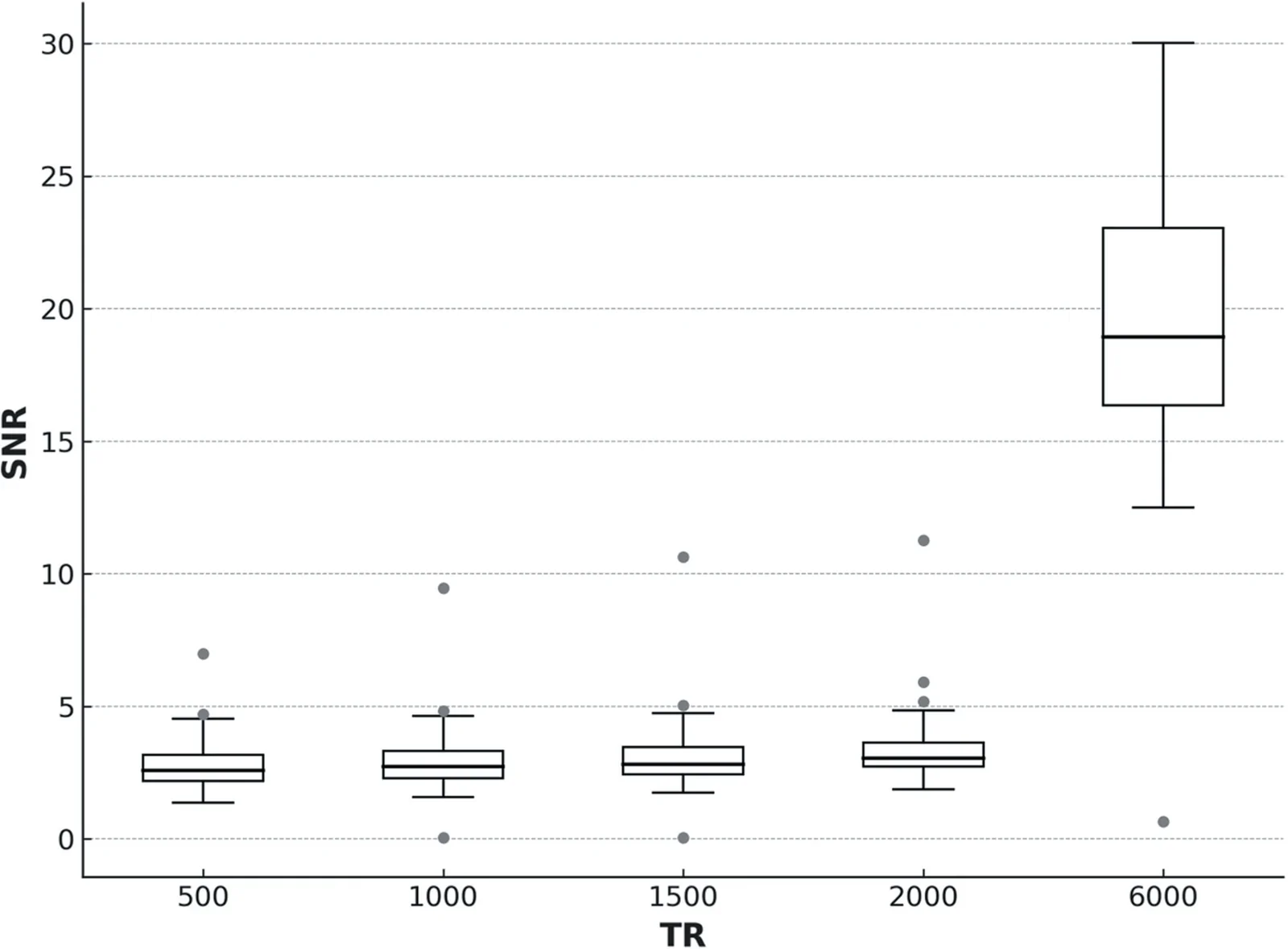
Box plot of aSNR by TR condition. Box plots illustrate the distribution of the aSNR obtained from DWI with b = 2000 s/mm^2^ at various TRs (TR = 500, 1000, 1500, 2000, and 6000 ms). The aSNR was calculated as the ratio of lesion SI to the standard deviation of background noise. The aSNR at TR6000 was significantly higher than that at TR500, TR1000, TR1500, and TR2000 (*P* < 0.001). Additionally, the aSNR at TR2000 was significantly higher than that at TR500 (*P* = 0.034). No statistically significant differences were observed among the aSNR values for TR500, TR1000, and TR1500. aSNR, apparent SNR; DWI, diffusion-weighted imaging; SI, signal intensity.

**Fig. 4:**
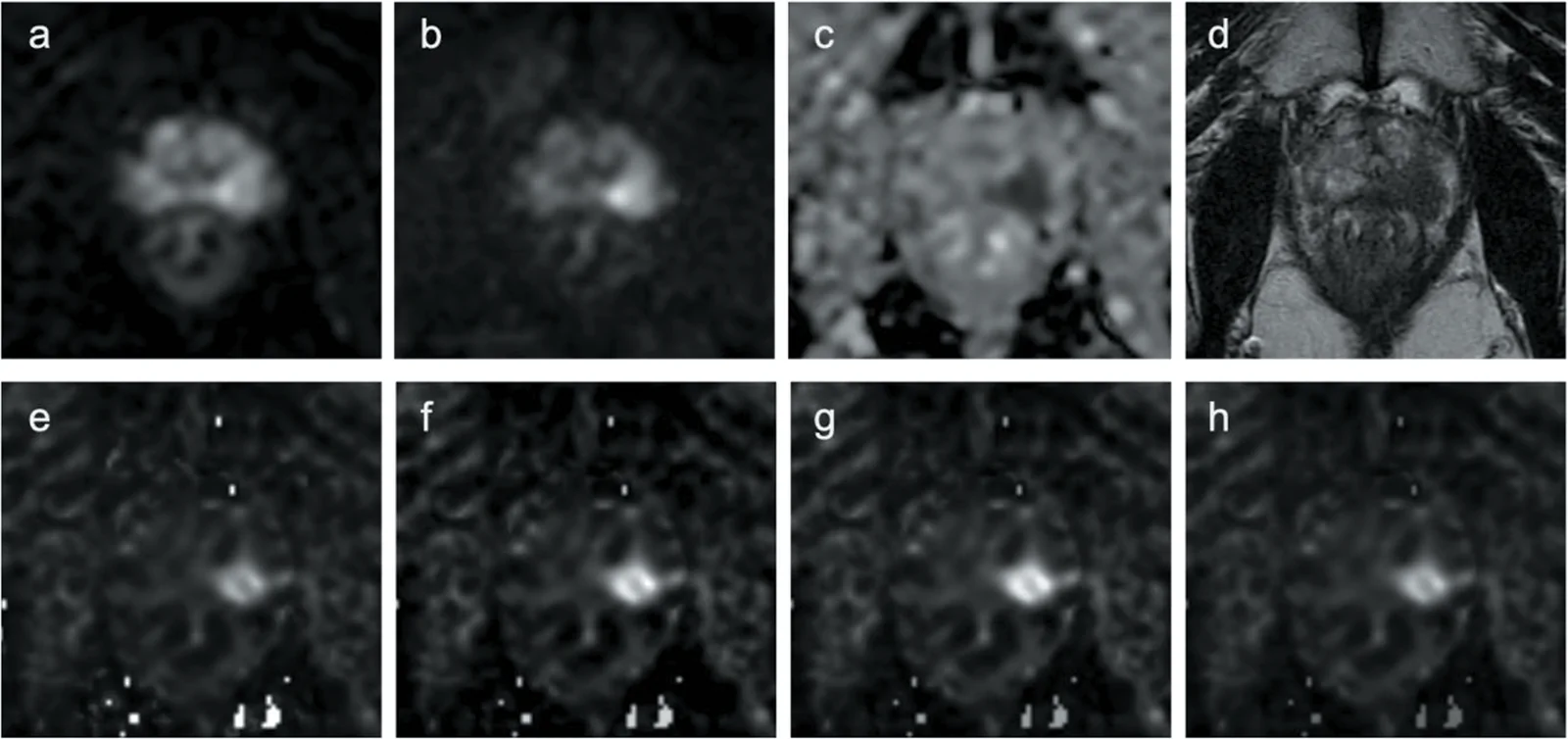
Comparison of conventional DWI and synthetic DWI with different TRs for prostate cancer evaluation. Images of a 68-year-old male patient with prostate cancer (Gleason score 4 + 3 = 7) in the left peripheral zone are shown. Conventional MRI sequences and synthetic DWI with varying TR values were used to assess the lesions. (**a**) Conventional DWI (b = 1000 s/mm^2^, TR = 6000 ms, TE = 70 ms): The tumor exhibits mild hyperintensity. (**b**) Conventional DWI (b = 2000 s/mm^2^, TR = 6000 ms, TE = 70 ms): The hyperintensity of the lesion is more pronounced compared to conventional  DWI (b = 1000), but parts of the lesion remain faint. (**c**) ADC map (b = 0–1000 s/mm^2^): The lesion shows a markedly decreased ADC value (ADC = 0.81 × 10^−3^ mm^2^/s). (**d**) T2WI: The lesion appears as a hypointense area. (**e**) Synthetic DWI (b = 2000 s/mm^2^, TR = 500 ms, TE = 0 ms): the lesion contrast is enhanced compared to conventional DWI, although slightly lower than that of TR = 1000 and 1500 ms. (**f**) Synthetic DWI (b = 2000 s/mm^2^, TR = 1000 ms, TE = 0 ms): The lesion demonstrates excellent diffusion contrast and is clearly visible. (**g**) Synthetic DWI (b = 2000 s/mm^2^, TR = 1500 ms, TE = 0 ms): Similar to TR = 1000 ms, the lesion contrast remained favorable. (**h**) Synthetic DWI (b = 2000 s/mm^2^, TR = 2000 ms, TE = 0 ms): Compared to TR = 1000 and 1500 ms, the lesion contrast was slightly reduced. Overall, synthetic DWI with TR of 1000 and 1500 ms provided the most favorable tumor contrast for prostate cancer detection. ADC, apparent diffusion coefficient; DWI, diffusion-weighted image; T2WI, T2-weighted image.

**Table 1:** Acquisition parameters for SyDWI and conventional DWI

	Conventional DWI	SyDWI	
b-value (s/mm^2^)	0, 1000, and 2000	0	1000 and 2000
FOV (mm^2^)	300 × 300	300 × 300	300 × 300
TR/TE (ms)	6000/70	6000/70	6000/70
		6000/140
		2000/70
Acquired voxel size (mm^3^)	3.13 × 3.13 × 3.00	3.13 × 3.13 × 3.00	3.13 × 3.13 × 3.00
SENSE reduction factor	2	2	2
NSA	2 (b0), 4 (b1000), 8 (b2000)	2 (b0)	4 (b1000) and 8 (b2000)
Scan time	3m 54s	4m 06s	

SyDWI, synthetic diffusion-weighted imaging generated by combining T1/T2 information (from multiple TR/TE scans at b = 0) with diffusion parameters from b = 1000 and 2000 s/mm^2^. SENSE reduction factor indicates parallel imaging acceleration. NSA, number of signal averages (repetitions). Acquired voxel size indicates in-plane resolution (3.13 × 3.13 mm^2^) × slice thickness (3.00 mm). Scan times are approximate and may vary depending on patient and system factors.

DWI, diffusion-weighted imaging; NSA, number of signal averages; SENSE, sensitivity encoding; synDWI, synthetic DWI.

**Table 2:** Baseline characteristics of patients and index lesions

Item	Value
Age (years), median (IQR)	69.5 (64.0–74.0)
T-PSA (ng/mL), median (IQR)	7.86 (5.46–11.04)
PSAD, median (IQR)	0.30 (0.24–0.48)
Location of lesion	
• Peripheral zone (PZ)	31 (55.4%)
• Transition zone (TZ)	22 (39.3%)
• PZ + TZ	3 (5.4%)
Tumor ADC (×10^−3^ mm^2^/s), median (IQR)	0.75 (0.68–0.84)
Tumor size (mm), median (IQR)	13.6 (9.1–17.9)
PI-RADS classification	
• PI-RADS 3	5 (8.9%)
• PI-RADS 4	32 (57.1%)
• PI-RADS 5	18 (32.1%)
• Not evaluable	1 (1.8%)
Pathological T stage	
• T2a	10 (17.9%)
• T2c	20 (35.7%)
• T3a	24 (42.9%)
• T3b	2 (3.6%)
Gleason score	
3 + 4 = 7	32 (57.1%)
4 + 3 = 7	18 (32.1%)
4 + 4 = 8	2 (3.6%)
4 + 5 = 9	4 (7.1%)

PSAD, T-PSA/prostate volume. Tumor size measured in mm on T2-weighted images (mean of longest axial diameter). Pathological T stage and Gleason score based on final pathology.

ADC, apparent diffusion coefficient; IQR, interquartile range; PSAD, prostate-specific antigen density; PI-RADS, Prostate Imaging Reporting and Data System; T-PSA, total prostate-specific antigen.

**Table 3:** Comparison of LCS, DWI score (PI-RADS v2.1), and prostate background signal across different TR conditions

Parameter	Reader	Kruskal–Wallis test	500 ms vs. 1000 ms	500 vs. 1500	500 vs. 2000	500 vs. 6000	1000 vs. 1500	1000 vs. 2000	1000 vs. 6000	1500 vs. 2000	1500 vs. 6000	2000 vs. 6000
LCS	Reader 1	*P* < 0.001	3.3 ± 0.6 vs. 3.8 ± 0.5*	3.3 ± 0.6 vs. 3.9 ± 0.4*	3.3 ± 0.6 vs. 3.8 ± 0.4*	3.3 ± 0.6 vs. 3.8 ± 0.4*	3.8 ± 0.5 vs. 3.9 ± 0.4	3.8 ± 0.5 vs. 3.8 ± 0.4	3.8 ± 0.5vs. 3.8 ± 0.4	3.9 ± 0.4 vs. 3.8 ± 0.4	3.9 ± 0.4 vs. 3.8 ± 0.4	3.8 ± 0.4 vs. 3.8 ± 0.4
	Reader 2	*P* < 0.001	3.4 ± 0.7 vs. 3.8 ± 0.4	3.4 ± 0.7 vs. 3.8 ± 0.4*	3.4 ± 0.7 vs. 3.8 ± 0.4*	3.4 ± 0.7 vs. 3.8 ± 0.5*	3.8 ± 0.4 vs. 3.8 ± 0.4	3.8 ± 0.4 vs. 3.8 ± 0.4	3.8 ± 0.4 vs. 3.8 ± 0.5	3.8 ± 0.4 vs. 3.8 ± 0.4	3.8 ± 0.4 vs. 3.8 ± 0.5	3.8 ± 0.4 vs. 3.8 ± 0.5
DWI score	Reader 1	*P* < 0.001	3.6 ± 0.6 vs. 3.9 ± 0.5	3.6 ± 0.6 vs. 3.9 ± 0.4*	3.6 ± 0.6 vs. 3.9 ± 0.5*	3.6 ± 0.6 vs. 3.9 ± 0.3*	3.9 ± 0.5 vs. 3.9 ± 0.4	3.9 ± 0.5 vs. 3.9 ± 0.5	3.9 ± 0.5 vs. 3.9 ± 0.3	3.9 ± 0.4 vs. 3.9 ± 0.5	3.9 ± 0.4 vs. 3.9 ± 0.3	3.9 ± 0.5 vs. 3.9 ± 0.3
	Reader 2	*P* < 0.001	3.7 ± 0.7 vs. 3.9 ± 0.4	3.7 ± 0.7 vs. 3.9 ± 0.4	3.7 ± 0.7 vs. 4.0 ± 0.4*	3.7 ± 0.7 vs. 3.9 ± 0.5*	3.9 ± 0.4 vs. 3.9 ± 0.4	3.9 ± 0.4 vs. 4.0 ± 0.4	3.9 ± 0.4 vs. 3.9 ± 0.5	3.9 ± 0.4 vs. 4.0 ± 0.4	3.9 ± 0.4 vs. 3.9 ± 0.5	4.0 ± 0.4 vs. 3.9 ± 0.5
Background signal	Reader 1	*P* < 0.001	3.7 ± 0.6 vs. 3.6 ± 0.6	3.7 ± 0.6 vs. 3.6 ± 0.6	3.7 ± 0.6 vs. 3.4 ± 0.6*	3.7 ± 0.6 vs. 1.1 ± 0.8*	3.6 ± 0.6 vs. 3.6 ± 0.6	3.6 ± 0.6 vs. 3.4 ± 0.6	3.6 ± 0.6 vs. 1.1 ± 0.8*	3.6 ± 0.6 vs. 3.4 ± 0.6	3.6 ± 0.6 vs. 1.1 ± 0.8*	3.4 ± 0.6 vs. 1.1 ± 0.8*
	Reader 2	*P* < 0.001	3.7 ± 0.6 vs. 3.8 ± 0.6	3.7 ± 0.6 vs. 3.8 ± 0.4	3.7 ± 0.6 vs. 3.8 ± 0.4	3.7 ± 0.6 vs. 1.4 ± 0.5*	3.8 ± 0.6 vs. 3.8 ± 0.4	3.8 ± 0.6 vs. 3.8 ± 0.4	3.8 ± 0.6 vs. 1.4 ± 0.5*	3.8 ± 0.4 vs. 3.8 ± 0.4	3.8 ± 0.4 vs. 1.4 ± 0.5*	3.8 ± 0.4 vs. 1.4 ± 0.5*

The numeric values in each cell represent the mean ± standard deviation of each parameter (LCS, DWI score, and prostate background signal) as assessed by the respective reader. Although these scores are ordinal data, mean values are presented to clarify the subtle differences between conditions.

Asterisks (*) indicate significant differences, as determined by the Mann–Whitney U test with Bonferroni correction (*P* < 0.005).

The Kruskal–Wallis test was used for overall comparisons among the 5TR conditions.

LCS score: 0 = low, 1 = isolated, 2 = slightly high, 3 = moderately high, 4 = very high.

DWI score in PI-RADS v2.1: 1–5.

Prostate background signal: 0 = very high, 1 = moderately high, 2 = mildly high, 3 = slightly high, and 4 = low. DWI, diffusion-weighted imaging; LCS, lesion conspicuity score; PI-RADS, Prostate Imaging Reporting and Data System.
